# Novel paddle stroke analysis for elite slalom kayakers: Relationship with force parameters

**DOI:** 10.1371/journal.pone.0192835

**Published:** 2018-02-28

**Authors:** Leonardo Henrique Dalcheco Messias, Filipe Antônio de Barros Sousa, Ivan Gustavo Masseli dos Reis, Homero Gustavo Ferrari, Claudio Alexandre Gobatto, Camila Caputo Saldanha Serra, Marcelo Papoti, Fúlvia Barros Manchado-Gobatto

**Affiliations:** 1 School of Applied Sciences, University of Campinas, Limeira, Sao Paulo, Brazil; 2 University of São Paulo, School of Physical Education, Bandeirantes, Ribeirão Preto, São Paulo, Brazil; Nanyang Technological University, SINGAPORE

## Abstract

This study was divided into two complementary parts. In Part 1, we proposed a novel paddle strokes analysis based on the force signal from a 30-s all-out tethered test; and compared these results with video recordings. In Part 2, we investigated the relationship between force data from the same test with paddle stroke results from both methods. Eleven male elite slalom kayakers (Brazilian national team) were evaluated. The tethered test was conducted for force parameters analysis (peak-force, mean-force, impulse). Video recording analysis was conducted, and the performed strokes (V.Number_Paddle_) was counted and frequency (V.Frequency_Paddle_) calculated by the V.Number_Paddle_ divided by 30 (i.e. total time of test). The new method consisted of performed strokes and frequency achievement from a load cell force signal analysis (S.Number_Paddle_ and S.Frequency_Paddle_, respectively). Paired test-t did not show difference between methods results, but significant correlations were only obtained for the number of paddle strokes. Force parameters were only correlated with S.Number_Paddle_ and S.Frequency_Paddle_. Overall, considering the theoretical and practical application, we propose that the new method should be used as an alternative to the video recording.

## Introduction

In kayaking, the force development during strokes has significant relevance for overcoming the aerodynamic and hydrodynamic resistances (i.e., drag), thus increasing the boat velocity [1]. Also, the influence of force on the athlete’s performance been demonstrated [2–4]. However, information on factors that could influence the total force developed, such as stroke types, techniques, and frequency, remain scarce, especially in canoe slalom discipline [5–8].

Dealing with these limitations, our group recently developed a specific on-water tethered ergometer to evaluate slalom kayakers [9]. The tethered canoe system (TCS) allows the application of physiological protocols concomitantly with force data acquisition. Furthermore, we demonstrated significant and inverse relationships between variables from a 30-s all-out test (e.g., peak force, mean force, and impulse) with the slalom kayakers performance (i.e., time of race) during a simulated canoe slalom race [9].

Despite the TCS progress and initial promising results, some points remain to be clarified. We believe the next step is to investigate which factors contribute to the force development during the tethered tests. Thus, this TCS’s 30-s all-out test must evaluate the number and frequency of paddle strokes. With this information in hands, coaches may analyze if the total force produced is related with the number and frequency of performed strokes. Since the total force produced during the TCS’s 30-s all-out test is related with the athletes performance during simulated race [9], information about the performed strokes and total force production may improve the slalom kayakers training sessions and, therefore, their performance. To our knowledge, this has not yet been addressed.

Conversely, investigations concerning the paddle stroke analysis (i.e., number and frequency) were conducted with other canoeing disciplines [3, 10, 11]. To generate paddle strokes and force development data, sophisticated equipment, including multiple cameras for the two-dimensional (2D) and three-dimensional (3D) kinematics analysis, are used. Despite generating important data from direct measurement, this equipment and analysis method might not be accessible to coaches during a daily routine. Although canoe slalom coaches could record training sessions and later analyze performed paddle stroke data, this procedure can be time-consuming.

Alternatively, accelerometers coupled to the paddle shaft can provide real-time information about the blade/water interaction [12], stroke cadence [13], and stroke power [14]. On the other hand, these studies were designed to provide alternative equipment to acquire the above-mentioned variables, and it is understandable that few subjects were considered in these investigations. However, taking into account the slalom athletes variability in terms of force development and anaerobic metabolism [5], new procedures must be conducted within a larger sample, mainly for elite athletes. Moreover, the association between stroke characteristics (i.e., number and frequency of performed strokes) and the total force produced remain scarce, especially for the canoe slalom. Previous reports have suggested that this association might influence the performance of slalom athletes [5, 15].

Therefore, in this study, we are proposing an alternative analysis for the evaluation of number and frequency of paddle strokes performed by slalom athletes during a 30-s all-out test. Moreover, we investigate the relationship between results provided by this analysis with total force produced during the same test. Thus, in Part 1, our aims were: a) to propose a new method based on the force signal for measuring the slalom kayakers paddle strokes during a 30-s all-out tethered test; and; b) to compare the new method results with the counted paddles strokes from the video recording. Since the new method is based on a direct measurement of total force signal, we hypothesized that similar results with the video recording method for number and frequency of performed strokes will be obtained. In Part 2, we investigated the relationship between the total force data from the all-out test with the paddle stroke results (i.e., the number of paddle strokes and frequency) from both methods. Considering the force signal method is based on a sensitive analysis of total force vector in a time series, we hypothesized that the number of paddle strokes/frequency would be significantly correlated with the total force parameters (peak force, mean force and impulse) obtained from the TCS’s 30-s all-out test.

## Materials and methods

### Design

All procedures were designed to address the hypotheses of this study. Eleven male slalom kayakers from the Brazilian national team were evaluated for a two-week period. Evaluations took place at approximately the same time of day (± 1 h). All subjects were in the pre-season training phase. Part 1 was conducted to compare the number and frequency of paddle strokes from a novel analysis based on the force signal from the 30-s all-out tethered test with those from the video recording. Subsequently, in Part 2, we related the results from Part 1 with the force parameters obtained from the 30-s all-out test.

#### Subjects

Eleven male elite slalom kayakers (19 ± 2-years-old, 71.0 ± 6.9 kg, 175.5 ± 7.6 cm, 10.2 ± 1.4 fat %) from the Brazilian national team (i.e., single kayak class—K-1) participated in this study. Fat percent was calculated according to Jackson and Pollock [16] by the measurement of seven skinfolds (i.e., chest, axilla, triceps, subscapular, abdomen, supra-iliac, and thigh). Ten of the eleven elite athletes (i.e., ~91%) are classified in the canoe slalom world ranking according to the International Canoe Federation (ICF). All athletes had at least 5 years of experience in international competitions. Athletes without international experience not familiarized within the K-1 class were not considered in the sample. Before the experimental procedures, athletes were asked to keep the same individual hydration/food habits and avoid hard physical activity, alcohol, and caffeine ingestion. Experimental procedures risks were previously explained, and athletes/parents provided written, informed consent authorizing the athlete’s participation in this study. All experiments were approved by the Ethics Committee of the School of Medical Sciences, University of Campinas.

### Procedures

All procedures were conducted on the Tethered Canoe System (TCS) [9], which was composed of a load cell (CSL/ZL-MK, SP, Brazil, 250 kgf capacity) using strain gauges from Wheatstone bridge application (1/2 Bridge). A keel (35 x 25 cm structure and 5.00 mm of thickness) was coupled bellow the boat rear to stabilize the ergometer. The load cell was fixed to a suction pad (Vonder, Curitiba, Brazil) anchored to the pool wall. In its center, it was coupled to a metallic hook connected to an elastic cord (length, 320 cm; external diameter, 16.60 mm; internal diameter, 4.00 mm; thickness, 6.30 mm; Altaflex, SP, Brazil). While some investigations preferred the use of inextensible steel cable for tethered swimming [17–18], running [19] or semi-tethered running [20] evaluations, others have used by the elastic cord, mainly for swimming [21–24]. The adoption of a steel cable instead an elastic cord is related to the characteristic of the ergometers. For instance, the steel cable was considered in the semi-tethered running to smooth variations of the orientation resulted from ground contact [20].

Our decision to opt for the elastic cord in the TCS is related to: a) the gradual resistance imposed by the elastic cord might represent some situations that occur during the canoe slalom competitions, such as the negotiation of upstream gates; b) the time taken to reach peak force during the all-out tethered test using an elastic cord is close to the mean time of gates transposition during slalom competitions [9], increasing the evaluation specificity; c) the total forces provided during the all-out test using the elastic cord are related to the slalom athlete’s performances from a simulated race [9]; d) due the relatively high forces applied, after a given paddle stroke, the inextensible steel cable pulls the slalom athlete a little backwards creating slack between paddle strokes, hampering the execution of the subsequent forward stroke; e) to analyze force decay along time, studies using the steel cable in tethered evaluation commonly employs digital filtering techniques [17–19]; on the other hand, the elastic cord serves as an analogic filter.

The digital signal was converted with a module USB 6008 (National Instruments, TX, USA). Signals were obtained at a high frequency of 1000 Hz, then processed and filtered using LabView-Signal-Express 2.0 (National Instruments, TX, USA). The system was calibrated with known weights (0, 5, 10, 15 and 20 kg) and converted into force units (N) using a linear equation (~R^2^ = 0.99).

All-out tests were accomplished in an outdoor swimming pool (25 m). Before the application of the 30-s all-out test, athletes performed two familiarization sessions. Each slalom kayaker used the same double-bladed paddle and the same boat (kayak model arrow, 355 cm length; 61 cm width, 16 kg mass). Although canoe slalom athletes use an individual set-up (i.e., boat and paddle) during competitions [5], we opted to standardize the TCS ergometer; otherwise, in future investigations, it will be difficult to replicate the results provided by the all-out tethered test. The kayak specifications used in the TCS (i.e., kayak length, width, and mass, as well as the double-bladed paddle) are in agreement with the rules of the International Canoe Federation (ICF). On the other hand, athletes used their individual paddle. Kayakers warmed up by self-paced paddling for 5 min, followed by a passive recovery of 5 min. The athletes were instructed to paddle for 10 s with the elastic cord under no strain, and then perform the test at an all-out intensity for 30 s after the signal (whistle) was sounded.

### Part 1—Comparison between the paddle stroke analyzing methods

The number of performed strokes and frequency were analyzed by two methods. The first was a video recording analysis using a Full HD video camera (Sony HDR-PJ200, 30× Optic Zoom, 300 Hz capture frequency) positioned on the swimming pool side edge. Each video record from the tests was individually analyzed and edited from the beginning (i.e., whistle sound) until the end of the test (i.e., next 30-s). Two experienced researchers analyzed the videos individually. A previous investigation [9] demonstrated that, during the all-out tethered test using the TCS slalom, athletes only performed forward strokes [6]. Therefore, the first researcher counted the performed forward strokes (V.Number_Paddle_) according to the following criteria: a) when blades move in the forward direction; b) when a propulsive stroke was performed within the top hands moving straight forward; and c) when paddle drags straight through the water [6]. The second researcher revised the videos to provide a double-check analysis on the performed strokes counted by the first. Both researchers agreed in all analysis, and a third researcher was not necessary. Also, the paddle frequency (V.Frequency_Paddle_) throughout the test was considered as: V.Frequency_Paddle_ = V.Number_Paddle_ / 30; and at each 5-s as: V.Frequency_Paddle5-s_ = V.Number_Paddle_ of each 5-s / 5.

**Fig 1:**
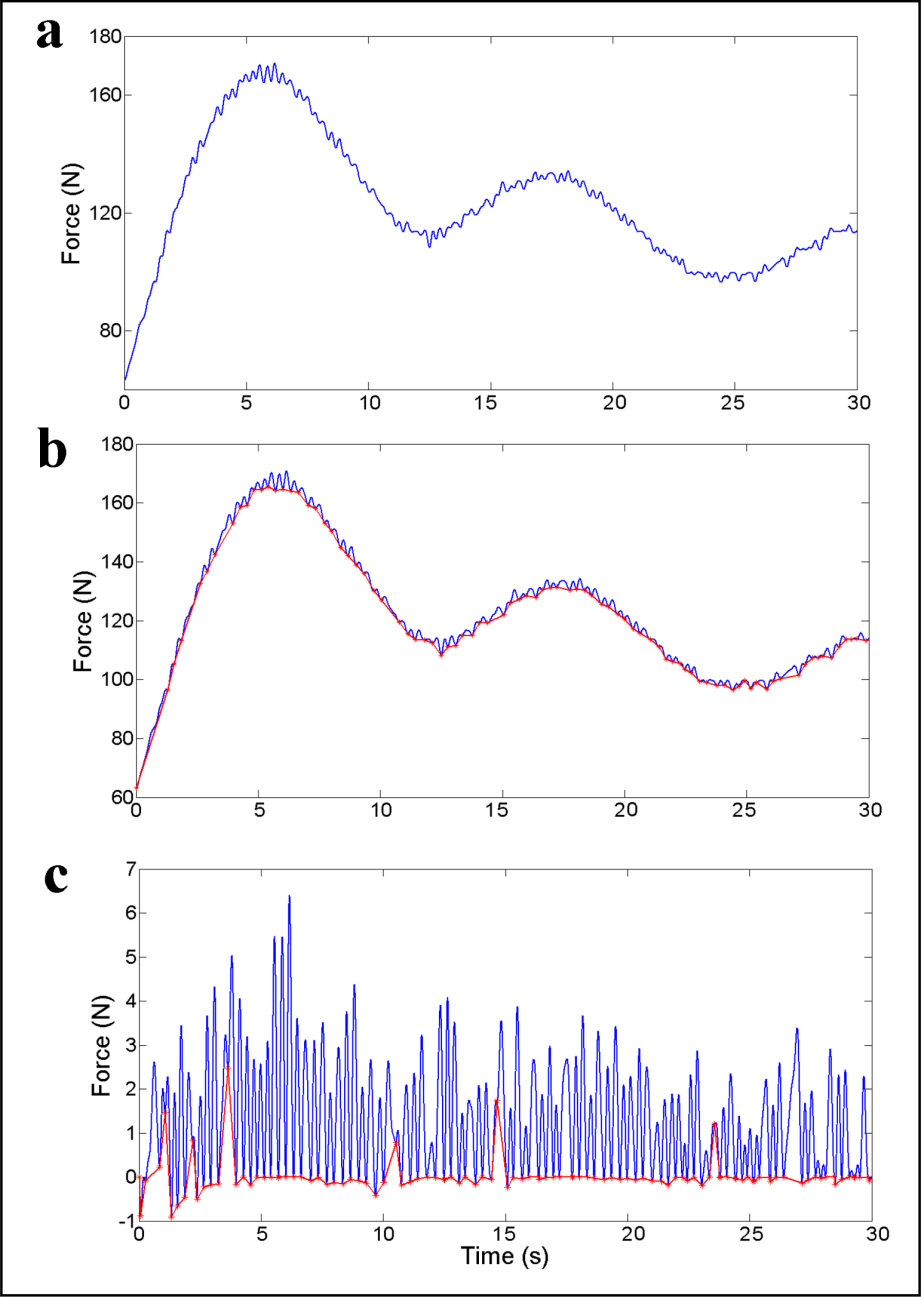
The moments when the force signal for a given athlete (a and b- black) changed from falling to rising were registered, and a new vector (PRV) was built with this information (b—gray). Then, by subtracting both vectors, a third vector was built (PRV2), and the oscillations become clearer (c). Finally, the moments when the force signal changed from falling to rising were registered again, being considered as a start and end of each paddle stroke.

The second method of stroke analysis relied on the total force signal measured by the load cell. The elastic cord used served as an analog filter, producing a semi-continuous signal (Fig 1). This method was based on the count of oscillations in the force vector in a time series, each force peak being defined as one paddle stroke. To identify each oscillation objectively, a MatLab (The MathWorks Inc., MA, USA) function was built (S2 File)).

At first, a derivative of the force vector was calculated to obtain its rate of change (RC). Then, the MatLab function identified every change of sign in the RC vector, building a new vector (change of sign vector—CSV) with the x,y coordinates of the force in the time series. Every time the RC vector positive change denotes the start of a rise in the force vector, defining the beginning of a new paddle stroke. The end of a stroke was defined as the start of the next one. Because of the elastic cord and the all-out characteristics of the test, in the test beginning (up until ~ 10 s), the force vector was always rising, so we interpolated the CSV vector linearly to achieve the same size of the force vector and then subtracted it from the force vector. Next, we used this difference to better identify the oscillations during the test, performing a new derivative of it, and again identifying the change of sign in the obtained RC, producing a new change of sign vector (CSV2) (Fig 1). The CSV2 vector was then used to obtain the number of paddle strokes (S.Number_Paddle_), and the individual stroke frequency (S.Frequency_Paddle_) for each given paddle stroke. Unlike V.Frequency_Paddle_, S.Frequency_Paddle_ was obtained by the time elapsed for completion of one cycle in CSV2, initiating at one down point (Fig 1C; red asterisks), passing a peak in the signal and finishing in the next down point. This enabled the calculation of frequency for each single paddle instead of a mean paddle frequency over the entire effort or a given period. A search for outliers was performed, excluding every paddle stroke that lasted less than 0.4 s. This period was adopted considering the unlikely capacity of the kayakers to perform more than 2 strokes per s. Therefore, we considered every signal <0.4 s as an odd noise from our system, possibly caused by double oscillations in one single paddle, or other oscillations caused by the kayak interaction to the water.

The number of performed strokes and frequency were also analyzed at 5-s intervals for both methods. Considering that strokes variables are expected to decay throughout the all-out test, with this procedure, we aimed to investigate whether these methods are reliable for the entire duration of the test. To address the first aim of this study, the number of performed strokes, frequency, and strokes variables at each 5-s from both methods were compared.

### Part 2—Relationship between the paddle strokes results with the force data

The paddle stroke results were tested for correlations with the force data from the 30-s all-out tethered test. These include the absolute and relative peak force, mean force, and impulse. The relative results were calculated based on the slalom kayaker body mass. The peak force (Peak_Force_) was considered as the highest force result registered during the all-out test. Mean force (Mean_Force_) was considered as the mean of the entire force signal. The impulse was calculated by the numerical integration of a trapezoidal method from the force signal total area.

### Statistical analyses

The statistical software package STATISTICA 7.0 (StatSoft, OK, USA) and the Matlab 5.3 software (MathWorks, Massachusetts, USA) were used for all analysis. The mean and standard deviation (s) were calculated for all studied variables. Homogeneity and normality were confirmed using the Levene and Shapiro-Wilk tests, respectively. Paired t-test, the coefficient of variation (CV), and effect sizes (ES) [25] were used to compare the variables from the video recording and the force signals. The Cohen’s categories used to evaluate the magnitude of the ES were: small if 0 ≤ |d|≤0.5; medium if 0.5 < |d| ≤ 0.8; and large if |d| > 0.8). The relationship analyses were conducted by the Pearson product moment correlation (r). The agreement between variables from the video recording and the force signals was conducted by the Bland-Altman analysis [26]. Confidence intervals [27] were calculated for the test-t statistical significance (p), CV, ES and the relationship analysis with α = 0.05(σ/√n) (i.e., 95%). In all cases, statistical significance was set at p<0.05.

## Results

### Part 1—Comparison between the paddle stroke analyzing methods

**Fig 2:**
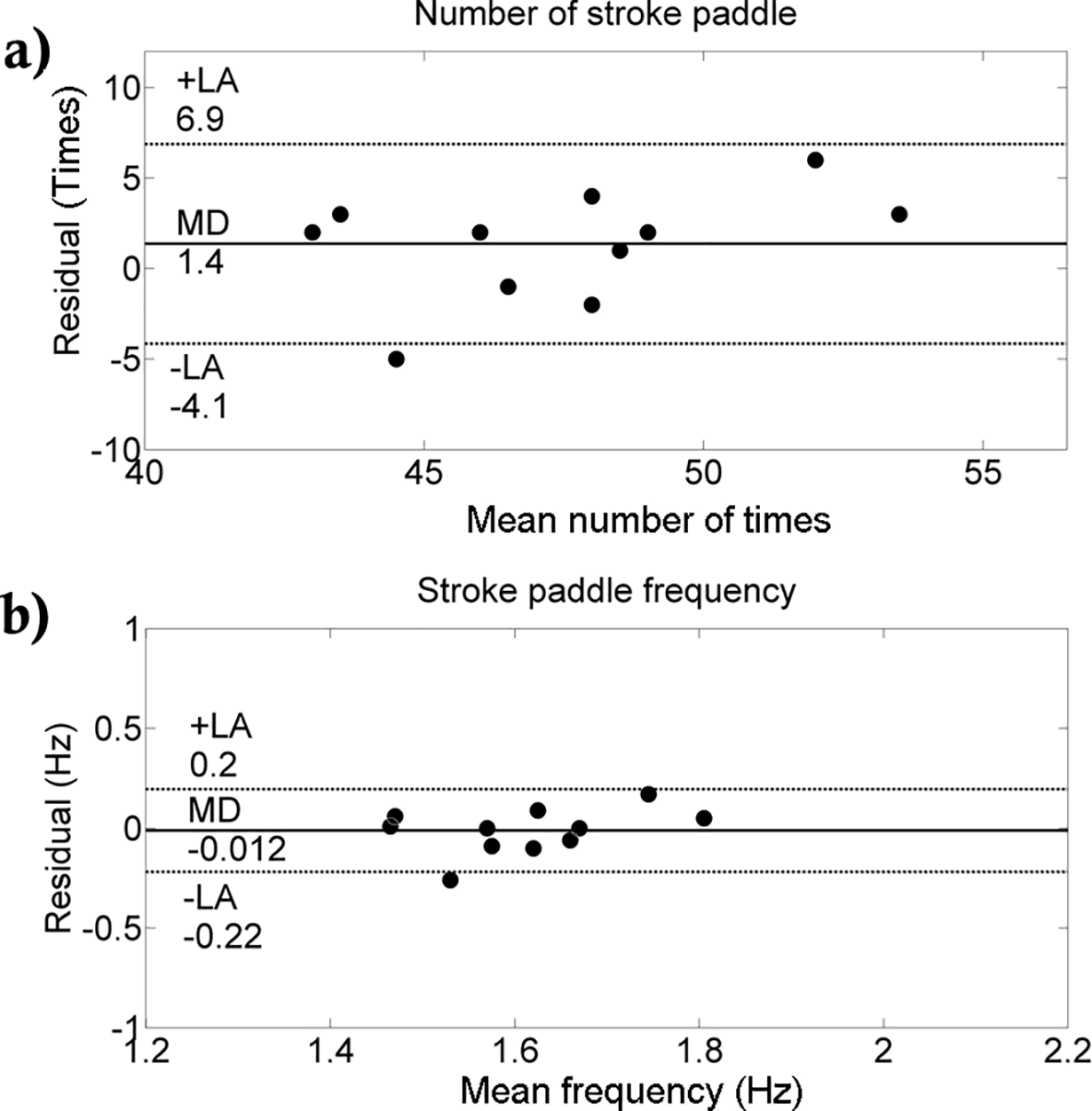
**Bland-Altman analysis between the number of paddle strokes (**a**) and frequency (**b**) from both methods**.

**Fig 3:**
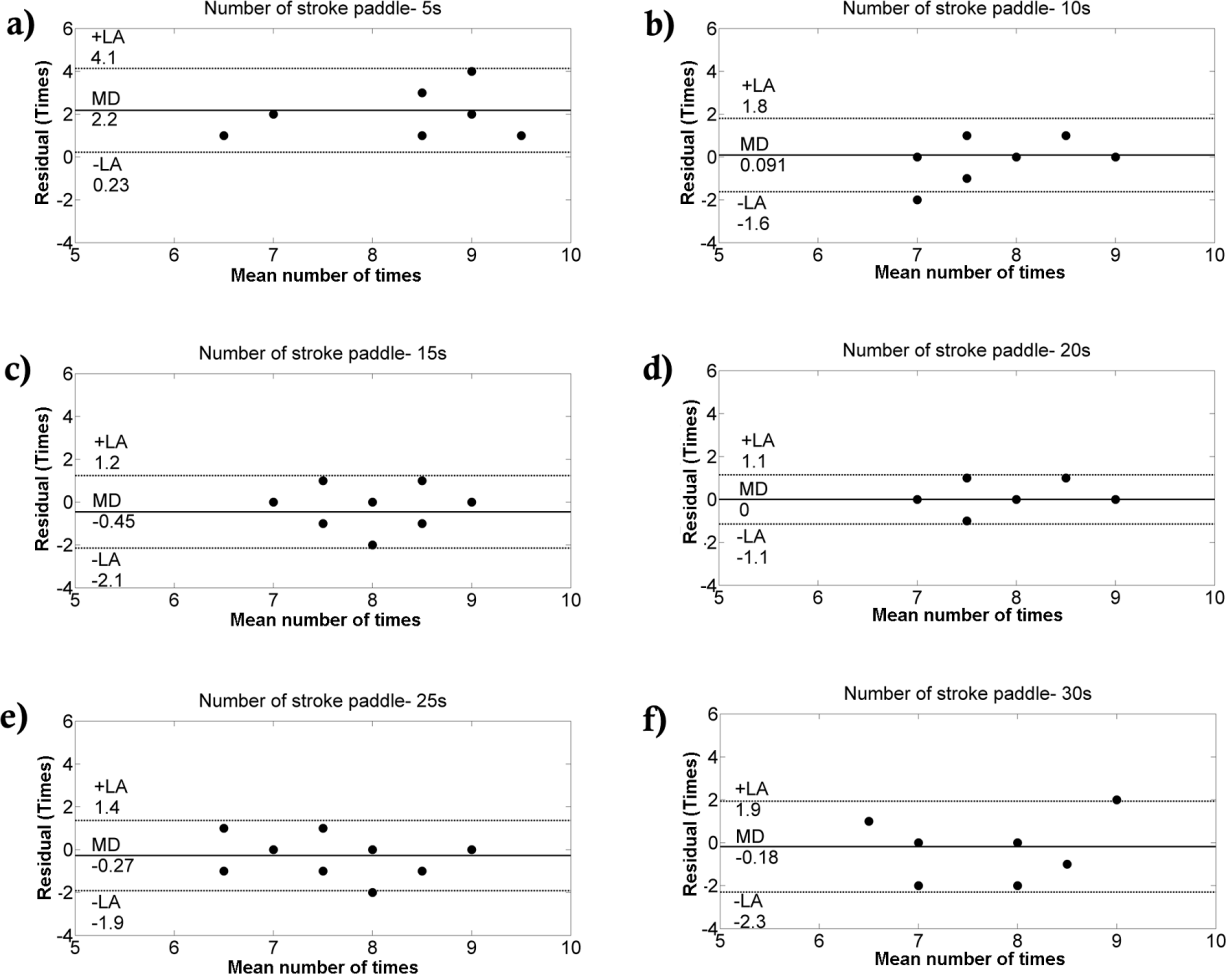
Bland-Altman analysis between the number of paddle strokes at each 5-s interval from both methods. Agreement between the performed strokes from 0 to 5 s **(a)**, 5 to 10 s **(b)**, 10 to 15 s **(c)**, 15 to 20 s **(d)**, 20 to 25 s **(e)**, and 25 to 30 s **(f)**.

**Fig 4:**
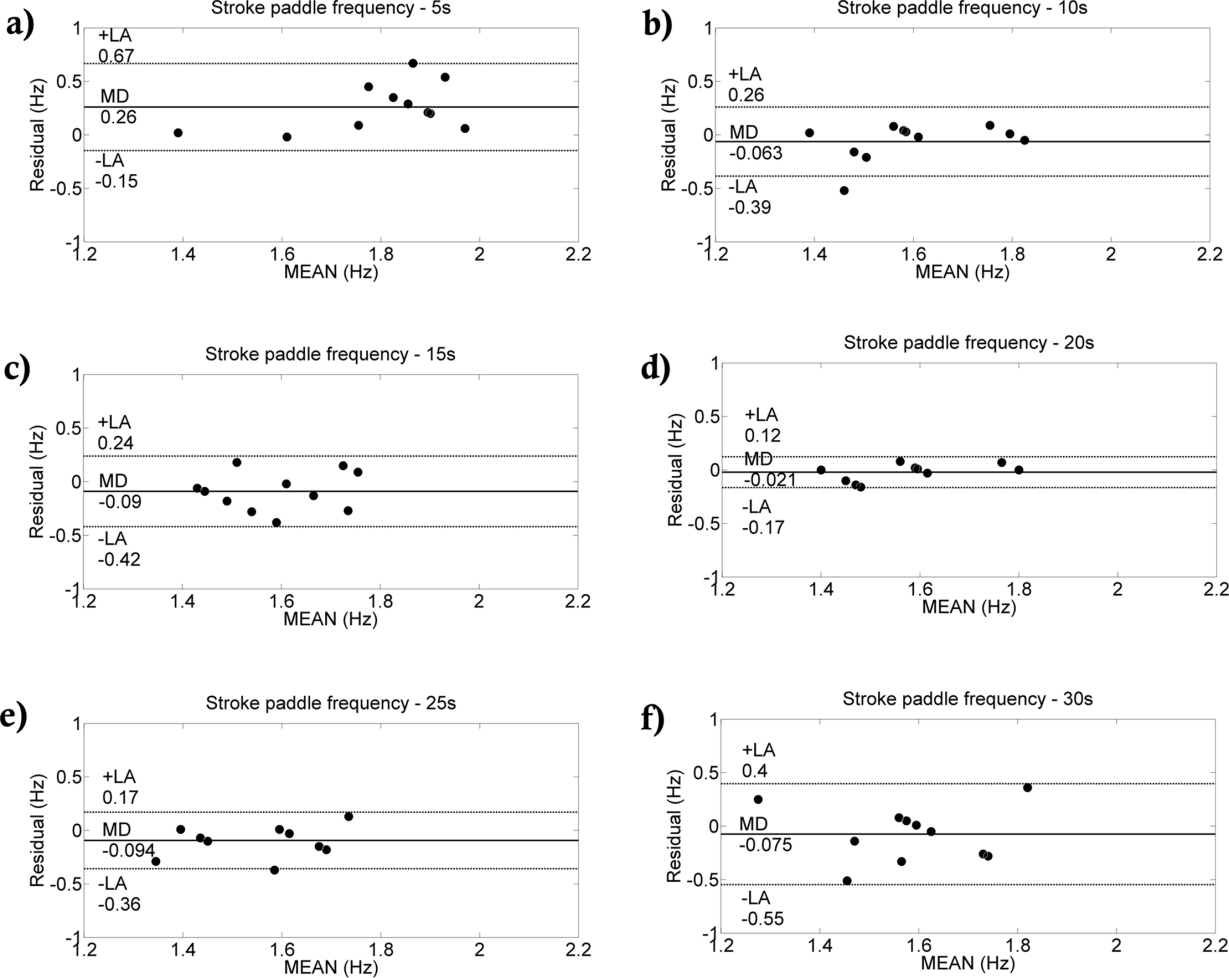
Bland-Altman analysis between paddle frequencies at each 5-s interval from both methods. Agreement between the paddle frequencies from 0 to 5 s **(a)**, 5 to 10 s **(b)**, 10 to 15 s **(c)**, 15 to 20 s **(d)**, 20 to 25 s **(e)**, and 25 to 30 s **(f)**.

**Fig 5:**
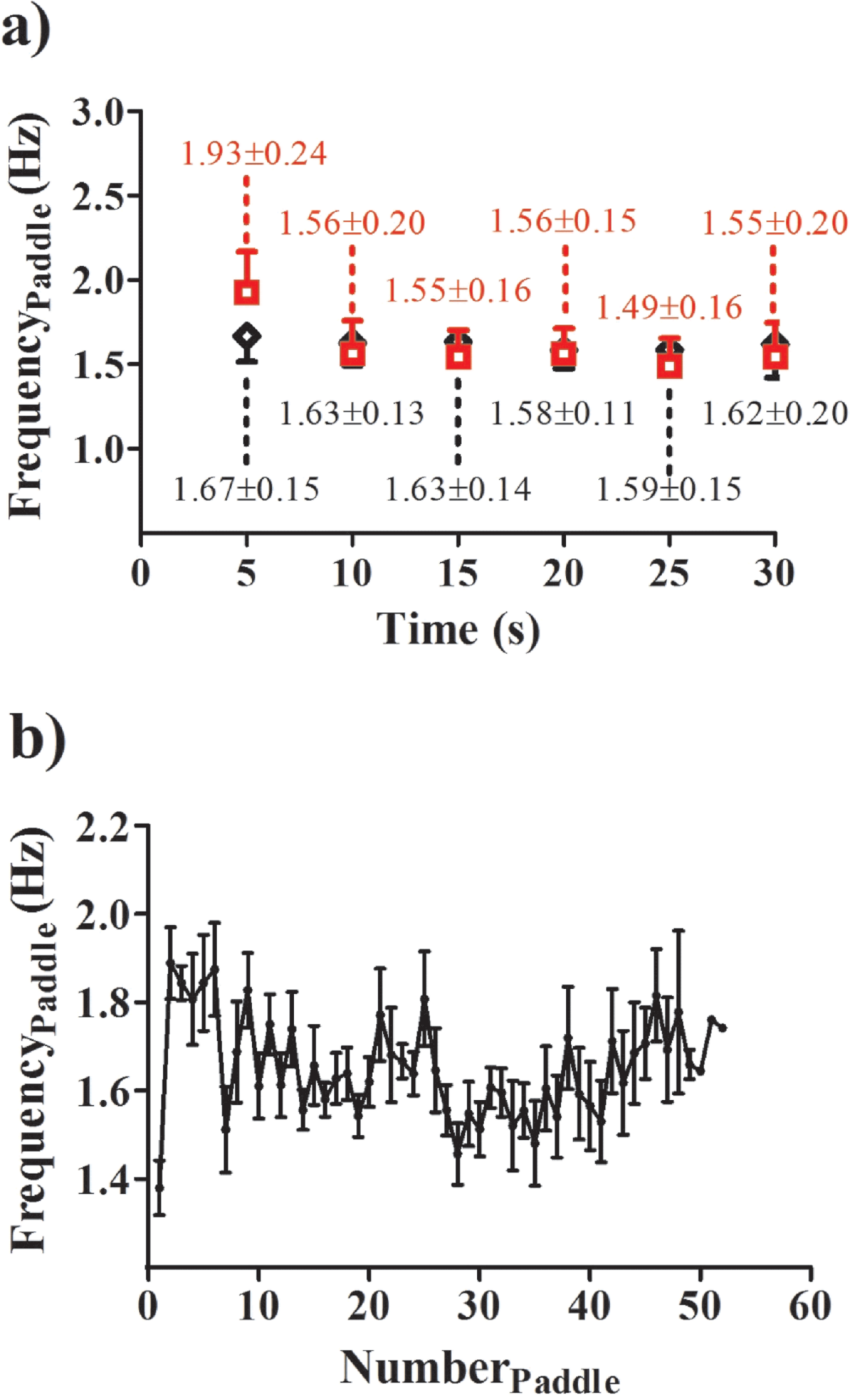
**a)–**Paddle frequency analysis from the video (red) and force signal method (black) considering the 5-s interval. **b)** Paddle frequency analysis conducted by means of the force signal method considering each paddle stroke performed by the slalom kayaker. All athletes performed at least 42 strokes. Only one slalom kayaker attained 52 strokes, explaining the absence of standard deviation on the last three points on the graph.

Comparing the two methods, we detected no differences in the number of performed paddle stroke and frequency (Table 1). A significant correlation was only obtained for the number of paddle strokes (r = 0.68; p = 0.02). Small and medium effect sizes were verified by the number of performed strokes and frequency (0.380 and 0.490, respectively). In general, low coefficient of variation was obtained (CV<10%). The Bland-Altman analysis between methods is shown in Figs 2, 3 and 4. The agreement between methods for the number of strokes is good (Fig 2A). Despite the fact that the agreement is weak during the first 5-s interval (Fig 3A), during the rest of the test, the agreement is very good (Fig 3B, 3C, 3D, 3E and 3F). Overall, good agreement was visualized for the paddle frequency (Figs 2B, 4A, 4B, 4C, 4D, 4E and 4F). The Fig 5 shows the paddle frequency analysis from both methods considering the 5-s intervals (5a) and by the force signal method at every stroke (5b).

**Table 1:** Comparison between the number of performed paddle stroke (Number_Paddle_) and frequency (Frequency_Paddle_) from the video recording (V) and the force signal (S).

	Mean ± s	p	r	ES	CV (%)
V.Number_Paddle_ (n)	48.18 ± 4.17	0.16	0.68*	0.380	4.53
	(2.91 ± 7.32)				
S.Number_Paddle_ (n)	46.82 ± 2.99				
	(2.09 ± 5.25)	(-0.2 ± 0.9)	(0.14 ± 0.91)	(0.10 ± 1.50)	(3.17 ± 7.95)
V.Frequency_Paddle_ (Hz)	1.61 ± 0.14	0.70	0.58	0.490	5.03
	(0.10 ± 0.25)				
S.Frequency_Paddle_ (Hz)	1.62 ± 0.10				
	(0.07 ± 0.18)	(-2.3 ± 3.3)	(-0.03 ± 0.88)	(0.01 ± 19.43)	(3.51 ± 8.83)

*Significant relationship; Lower and upper confidence limits are showed between parentheses; p = paired test-t statistical significance; r = Pearson product moment; ES = effect sizes; CV = coefficient of variation; V.NumberPaddle−number of performed strokes acquired by the video recording method; S.NumberPaddle−number of performed strokes acquired by the force signal method; V.FrequencyPaddle−analysis of paddle frequency performed by the video recording method; S.FrequencyPaddle−analysis of paddle frequency performed by the force signal method; The present analysis was performed for the total time of the test (i.e 30-s).

### Part 2—Relationship between the paddle strokes results with the force data

The absolute and relative peak force, mean force, and impulse are shown in Table 2. Low coefficients of variation were obtained for all force results. Positive and significant correlations were visualized between all force data (i.e., absolute and relative peak force, mean force, and impulse) with the S.Number_Paddle_ and S.Frequency_Paddle_ (range = 0.74–0.83) (Table 3). On the other hand, poor relationships were obtained between the results from the video recording (i.e., V.Number_Paddle_ and V.Frequency_Paddle_) with the force data (range = 0.31–0.41).

**Table 2:** Absolute (A) and relative (R) force data from the 30-s tethered all-out test.

	A.Peak_Force_	A.Mean_Force_	R.Peak_Force_	R.Mean_Force_	A.Impulse	R.Impulse
	(N)	(N)	(N•kg^-1^)	(N•kg^-1^)	(N•s)	(N•s•kg^-1^)
Mean	178.21	126.35	2.52	1.79	3766.05	53.33
s	27.21	18.43	0.37	0.26	585.72	8.27
CI–s (α = 0.05)	19.01 ± 47.75	12.88 ± 32.34	0.26 ± 0.65	0.18 ± 0.46	409.25 ± 1027.90	5.78 ± 14.51
CV (%)	5.55	5.86	5.78	5.99	5.43	5.45
CI–CV (α = 0.05)	3.93 ± 9.42	4.15 ± 9.95	4.09 ± 9.81	3.93 ± 9.42	4.24 ± 10.17	3.86 ± 9.25

**CI**–Lower and upper confidence limits; **CV**–coefficient of variation.

**Table 3:** Relationship between the paddle strokes results and the force data.

	V.Number_Paddle_ (n)	S.Number_Paddle_ (n)	V.Frequency_Paddle_ (Hz)	S.Frequency_Paddle_ (Hz)
**A.Peak**_**Force**_	r = 0.34; p = 0.30	r = 0.76*;p = 0.01	r = 0.34;p = 0.30	r = 0.81*;p = 0.001
**(N)**	CI = -0.33–0.78	CI = 0.29–0.93	CI = -0.33–0.78	CI = 0.41–0.95
**R.Peak**_**Force**_	r = 0.31;p = 0.34	r = 0.77*;p = 0.001	r = 0.31;p = 0.34	r = 0.79*;p = 0.001
**(N•kg**^**-1**^**)**	CI = -0.36–0.77	CI = 0.32–0.94	CI = -0.36–0.77	CI = 0.36–0.94
**A.Mean**_**Force**_	r = 0.41;p = 0.19	r = 0.79*;p = 0.001	r = 0.41;p = 0.19	r = 0.83*;p = 0.001
**(N)**	CI = -0.25–0.81	CI = 0.36–0.94	CI = -0.25–0.81	CI = 0.46–0.95
**R.Mean**_**Force**_	r = 0.38;p = 0.24	r = 0.78*;p = 0.001	r = 0.38;p = 0.24	r = 0.80*; p = 0.001
**(N•kg**^**-1**^**)**	CI = -0.28–0.80	CI = 0.34–0.94	CI = -0.28–0.80	CI = 0.38–0.95
**A.Impulse**	r = 0.37;p = 0.25	r = 0.75*;p = 0.01	r = 0.37;p = 0.25	r = 0.80*; p = 0.001
**(N•s)**	CI = -0.30–0.79	CI = 0.27–0.93	CI = -0.30–0.79	CI = 0.38–0.95
**R.Impulse**	r = 0.33;p = 0.31	r = 0.74*;p = 0.01	r = 0.33;p = 0.31	r = 0.75*;p = 0.01
**(N•s•kg**^**-1**^**)**	CI = -0.34–0.78	CI = 0.25–0.93	CI = -0.34–0.78	CI = 0.27–0.93

Lower and upper confidence limits for Pearson product moment (r).

## Discussion

The present study demonstrate the possibility of gathering slalom kayaker’s paddle stroke data using a novel direct method. This method has several advantages compared with the video recording approach. These include the measurement of the number of performed paddle strokes and frequency using a time-saving and simple practical approach. Despite the similar results obtain by the two tested methods, significant relationships with the force data were only visualized using the new method. Thus, considering the theoretical basis and practical application, the force signal method provides better results than the video recording, and therefore, should be adopted.

Independently of the variability in racecourses and obstacles (e.g., gate placement, depth of water, and magnitude of waves) [28], slalom kayakers must perform high-intensity efforts (i.e., paddle strokes) to achieve better performance [5]. In line with this, Vieira et al., [15] have demonstrated the reproducibility of technical parameters during simulated races. Apart from the myriad of stroke types analyzed, these authors found that the total number of paddle strokes during the two races were not different, were significantly correlated, and provided a low coefficient of variation (p = 0.37; r = 0.81; CV = 4.53). Also, the stroke types performed by slalom kayakers during races and their effects on the boat were described in detail [6]. These studies have shown that paddle stroke data is critical for improving the performance of canoe slalom athletes, and must be properly investigated.

Since our group has shown a significant relationship between slalom kayaker’s performance and force parameters from the 30-s all-out test [9], we aimed to investigate the relationship between the number and frequency of performed paddle strokes during this test with the total force development. However, before we deal with this issue, another concern regarding the methods to collect paddle stroke data was investigated. Thus, considering the V.Number_Paddle_ and S.Number_Paddle_, our results suggest the number of paddle strokes performed during this test can be achieved from both methods. The agreement between the tested methods is weaker for the number of paddles in the first 5-s (Fig 3A). In the rest of the all-out test, the agreement between the two tested methods is good. Differences in the number of strokes could be explained by human bias when analyzing video footage, as well as the difficulty in defining the criteria for categorizing paddle strokes as in a given period. Regarding the signal method, at the beginning of the test, it is harder to identify the strokes, since the force is rapidly rising, explaining the greater divergence between the tested methods in the first 5s. Overall, when calculating the number of strokes, the agreement between the tested methods is good.

In addition to the number of paddle strokes performed, a non-significant relationship between the V.Frequency_Paddle_ and S.Frequency_Paddle_ (r = 0.58; p = 0.06) was also detected. Using the video recording, biases might be more pronounced when calculating the paddle frequency since this is a mean frequency over a given period (30-s in this case). For the analysis at each 5-s interval, a similar procedure was applied (i.e., the paddle strokes performed at each 5-s interval was divided by 5). For example, the first and third columns of Table 3 (V.Number_Paddle_ and V.Frequency_Paddle_) the correlations have similar p values and CI values. This problem is explained by the calculation used to obtain the paddle frequency throughout the test when using the video method since the paddle frequency was indirectly calculated as the number of paddle strokes divided by the time of the all-out test (e.g., 30-s). We propose that is it more difficult to calculate the paddle frequency using a video record than using the novel method proposed.

Conversely, the same data calculated by the force signal was conducted considering the specific time of a given paddle stroke. By proposing this method, we solve one of the limitation associated with the video recording approach related with the necessity of count the performed strokes. It is true that kinematic analysis from 2D and 3D video analysis allows a more detailed investigation of paddle stroke phases and frequency [1]. However, these procedures might not be practically applicable during the daily routine of canoe slalom training, thereby slowing the transition from theory to practice. Additionally, video methods are often time-consuming, technically challenging, and costly [4]. Although rate watches and phones are valid real-time measurements, these device still requires force development. The new method proposed here is relatively cheap (compared to 2D and 3D video analysis) and can be easily carried out immediately after the test, reducing the time spent on paddle stroke analysis. Furthermore, positive and significant relationships were visualized between all force variables from the all-out test with the slalom kayakers paddle frequency when analyzed by the force signal method.

Reinforcing the differences between stroke frequencies variables obtained by the two studied methods, significant relationships between the force and impulse parameters were only detected with the S.Number_Paddle_ and S.Frequency_Paddle_ (Table 3). The absence of significant relationships between force and impulse with V.Number_Paddle_ and V.Frequency_Paddle_ leads us to believe that the new method is more robust at identifying the number and frequency of paddle strokes performed during the test. Regarding practical application, it is hypothesized that a stronger athlete could develop faster paddle strokes and, thus, a higher number and frequency of paddle strokes. Although deliberately trying to accelerate the stroke frequency could result in faster but weaker strokes, we believe this was not the case in this study due to the level of the studied athletes. Thus, our results partially support this hypothesis. To further test this, future studies should address this hypothesis using other force/power meters that have been proposed for sprint [12] and slalom [14] kayaking. These studies can also compare the use of individual or generalized set-ups (paddle, blade, and kayak), since the blade-shape (for instance) might result in different efficiencies and paddling techniques [13]. An on-water analysis system to quantify force and other characteristics of kayak athletes has been proposed by Aitken and Neal [29]. Using this system, Aitken and Neal provided data on the paddler’s average force and impulse (for instance) based on force-time curves. Other systems based on force sensors attached to the bottom of the blade [12] or accelerometers [13] and strain gauges [14] attached near the hand position have also been proposed. These investigations have provided important information regarding the blade/water interaction [12], paddling cadence [13], and paddle force/power [14].

However, the results provided by this study share unique characteristics, such as: a) the force parameters provided by our system have been significantly correlated with aerobic [30] and anaerobic [9] variables, and with the kayakers’ performances [9, 30]; b) in addition to the comparison between the force parameters and paddle stroke results, we also compared the results from the TCS with those from the video recording; c) the TCS and the analysis proposed in this study was developed specifically for canoe slalom, which is also absent in the current literature; d) the perspectives proposed in this study were conducted with a larger sample that included the highest qualified athletes from Brazil; and e) the proposed system also enable real-time force analyses, we also provided a function provided as a supplementary material that enables it.

Among the natural obstacles encountered during races, the upstream gates are considered the most challenging for slalom kayakers [31]. Hunter [31] has suggested that the paddling technique used to negotiate upstream gates is similar between canoe and kayak classes. This author also demonstrated that faster athletes are better able to negotiate upstream gates. Although in these situations the resistance offered to the blade is low (considering the water phase of the stroke), it remains challenging because the drag of the kayak hull is high. Thus, once the significant relationship between peak force with S.Number_Paddle_ and S.Frequency_Paddle_ has been demonstrated, training sessions for peak force improvement may possibly enhance the athlete’s performance when negotiating upstream gates. Slalom kayakers must also overcome other factors, such as a great number of downstream gates, rocks, and water waves. These challenges require great force maintenance within a short period. In line with this, we detected significant relationships between mean force and impulse with S.Number_Paddle_ and S.Frequency_Paddle_. These data would be useful during training sessions, helping to improve the slalom kayaker’s performance. Furthermore, Zamparo et al., [32] have shown great aerobic participation during canoe slalom races (i.e., 49%), corroborating with the force maintenance improvement to achieve better results during canoe slalom races.

Despite recent reports from our group addressing various issues related to the canoe slalom [9, 30, 33–34], no study has investigated the relevance of slalom kayaker’s paddle strokes on total force development. To address this, here we describe a significant relationship between the force parameters with both the number of paddle strokes and stroke frequency. These data provide insights into race strategy and training approaches. Force development might be relevant to negotiating gates and transposing natural obstacles within less time during canoe slalom races, resulting in a better performance [5]. However, future studies should address limitations with the present investigation. For example, we do not provide an analysis of the force production during strokes made on the left or right side. Moreover, the results provided in our investigation are intended to the K-1 class; an adaptation of the TCS is required to the analysis of other classes, such as the C-1 (i.e., canoe single).

Overall, we conclude that the force signal from the 30-s all-out tethered test can be used as a new direct method for recording slalom kayaker’s paddle stroke data. Furthermore, we have shown that the number of paddle stroke and frequency performed during the all-out test are related to the force parameters obtained from the same test. These significant relationships were only detected when using the force signal method, suggesting that our new method (rather than the video method) should be used by coaches to improve slalom kayaker’s performance.

## Supporting information

S1 File Datasheet with all data used in the manuscript. Folder “Paddle Stroke Analysis” contains the data regarding the number and frequency of paddles performed during the 30-s all-out tethered test. In addition, the data states the analysis from the video and signal methods. Folder “Forces” states de absolute and relative peak force, mean force and impulse from the 30-s all-out tethered test.
(M) Click here for additional data file.

S2 File Matlab function. Matlab Function built to identify each oscillation objectively in the force vector in a time series. Each force peak is defined as one paddle stroke.
(XLSX)
